# Methyllysine Reader Plant Homeodomain (PHD) Finger Protein 20-like 1 (PHF20L1) Antagonizes DNA (Cytosine-5) Methyltransferase 1 (DNMT1) Proteasomal Degradation*

**DOI:** 10.1074/jbc.M113.525279

**Published:** 2014-02-03

**Authors:** Pierre-Olivier Estève, Jolyon Terragni, Kanneganti Deepti, Hang Gyeong Chin, Nan Dai, Alexsandra Espejo, Ivan R. Corrêa, Mark T. Bedford, Sriharsa Pradhan

**Affiliations:** From ‡ New England Biolabs Inc., Ipswich, Massachusetts 01938 and; the § Department of Molecular Carcinogenesis, University of Texas M.D. Anderson Cancer Center, Smithville, Texas 78957

**Keywords:** DNA-binding Protein, DNA Methylation, DNA Methyltransferase, Protein Degradation, Protein Methylation, MBT Domain, PHF20L1, SET7, UNC1215

## Abstract

Inheritance of DNA cytosine methylation pattern during successive cell division is mediated by maintenance DNA (cytosine-5) methyltransferase 1 (DNMT1). Lysine 142 of DNMT1 is methylated by the SET domain containing lysine methyltransferase 7 (SET7), leading to its degradation by proteasome. Here we show that PHD finger protein 20-like 1 (PHF20L1) regulates DNMT1 turnover in mammalian cells. Malignant brain tumor (MBT) domain of PHF20L1 binds to monomethylated lysine 142 on DNMT1 (DNMT1K142me1) and colocalizes at the perinucleolar space in a SET7-dependent manner. PHF20L1 knockdown by siRNA resulted in decreased amounts of DNMT1 on chromatin. Ubiquitination of DNMT1K142me1 was abolished by overexpression of PHF20L1, suggesting that its binding may block proteasomal degradation of DNMT1K142me1. Conversely, siRNA-mediated knockdown of PHF20L1 or incubation of a small molecule MBT domain binding inhibitor in cultured cells accelerated the proteasomal degradation of DNMT1. These results demonstrate that the MBT domain of PHF20L1 reads and controls enzyme levels of methylated DNMT1 in cells, thus representing a novel antagonist of DNMT1 degradation.

## Teaser

**Background:** SET7 monomethylates DNMT1, promoting its proteasomal degradation, yet methylated DNMT1 still remains throughout the cell cycle.

**Results:** The methyllysine reader PHF20L1 stabilizes methylated DNMT1. Disruption of PHF20L1 induces DNMT1 degradation and genome hypomethylation.

**Conclusion:** PHF20L1, an epigenetic reader, cooperates with writer and eraser to regulate epigenetic inheritance.

**Significance:** PHF20L1 can be targeted as a means of regulating DNMT1 activity and DNA methylation in cells.

## Introduction

Methylation at the 5-position of cytosine to generate 5mC^3^ on DNA represents a major epigenetic mechanism of gene silencing (1–3). 5mC occurs mostly at CpG dinucleotides and a small percentage at CHG and CHH sequences (4). In mammalian cells, there are three catalytically active DNA (cytosine-5) methyltransferase families represented by DNMT1, DNMT3A, and DNMT3B along with a methyltransferase-like protein, DNMT3L, which lacks a catalytic domain (5). The physical interaction and functional partnership between *de novo* methyltransferase DNMT3A, DNMT3B, and DNMT3L is well documented and is hypothesized to bind and methylate chromatin as a substrate (6, 7). Hypomethylation of the genome leads to chromosome instability, and aberrant DNA methylation is frequently observed in cancer (8, 9). Among the DNA methyltransferases, DNMT1 is known as the maintenance methyltransferase. DNMT1 preserves epigenetic inheritance by methylating the newly synthesized daughter strand during DNA replication (10). There are several interacting proteins of DNMT1, most notably PCNA and UHRF1 (ubiquitin-like containing PHD and RING finger domains 1) (11, 12). Both PCNA and UHRF1 are colocalized with DNMT1 during DNA replication, and deletion of UHRF1 by genetic knockout resulted in a severe loss (>80%) of 5mC in the embryonic stem cells. This suggests that there are proteins other than DNA methyltransferases that play an important role in the mechanism of epigenetic inheritance, perhaps via selective targeting of enzymes. Furthermore, UHRF1 has E3 ubiquitin-protein ligase activity that mediates the ubiquitination of target proteins, such as histone H3 and promyelocytic leukemia protein and alters gene expression (13, 14).

Another mechanism that influences epigenetic inheritance is the availability and stability of DNA methyltransferases in cells. Some mechanistic based inhibitors of DNMTs, such as 5-azacytidine, are a chemical analog of the cytosine nucleoside of DNA and RNA (15). 5-Azacytidine is thought to inhibit DNA methyltransferases at low doses, causing hypomethylation of DNA (16). Incorporation of 5-azacytidine into DNA leads to its covalent binding and capturing of DNA methyltransferases, resulting in the depletion of the enzyme pool in the cell (17, 18). In another study, 5-azacytidine and 5-aza-CdR treatment of cultured mammalian cells led to rapid degradation of DNMTs by the cellular proteasomal pathway (19). Therefore, it is plausible that both covalent attachment of these enzymes to DNA and their degradation may lead to poor maintenance of DNA methylation, consequently leading to hypomethylation of the genome and cellular apoptosis.

There are several studies supporting the role of lysine methylation of non-histone proteins in the regulation of protein activity and stability, particularly p53, ERα, RelA, and DNMT1 (20). Both p53 and ERα are stabilized by lysine methylation, whereas RelA and DNMT1 are destabilized. The effect of lysine methylation on histones is well studied, and it can be repressive or activating, depending on what lysine is methylated in a chromatin context (21). Indeed, distinct methyllysine marks recruit different reader proteins, resulting in different transcriptional responses. For example, histone H3K9me recruits HP1 proteins for gene silencing (22). Several protein domains are capable of recognizing methylation marks, including ANK repeats, WD40, plant homeodomain, PWWP, chromodomain, and MBT domain (23–25). Proteins containing MBT domains are products of polycomb group genes and have been implicated in transcriptional repression of developmental genes. Indeed, misregulation of MBT-containing proteins has been linked to various disease phenotypes (26).

In a previous report, we demonstrated that SET7 monomethylation of DNMT1 (DNMT1K142me1) leads to its proteasome-mediated protein degradation, and we have hypothesized that the antagonist LSD1 (lysine-specific demethylase 1) may prevent this degradation by removing the methyl mark (27). Furthermore, DNMT1K142me1 acts as an antagonist to Ser-143 phosphorylation, thus offering a methyl-phospho switch status that operates during the cell cycle. Phosphorylated DNMT1 is more stable than the methylated enzyme, and it is more abundant during the DNA synthesis stage of the cell cycle (28). Although the methylated species of DNMT1 accumulates during the late DNA synthesis stage and decreases thereafter, a significant percentage still remains throughout the whole cell cycle. This prompted us to search for a possible reader(s) of lysine-methylated DNMT1 and its role in the cell. In this report, we discuss a novel DNMT1K142me1 reader, PHF20L1, a protein that contains an active MBT domain (24). We studied its role in DNMT1 stability, loading, and epigenetic inheritance in mammalian cells.

## EXPERIMENTAL PROCEDURES

### 

#### 

##### Cell Treatments, Cell Cycle Synchronization, Protein Stability, Immunoprecipitation, GST Pull-down, Far-Western Blot, and Immunofluorescence

All cell lines (HeLa, COS-7, Jurkat, HCT116, and HEK293) were grown as per ATCC recommendations. Nuclear cell extracts were immunoprecipitated as described previously (29, 30). GST pull-downs and immunofluorescence studies were performed as described previously (30, 31). Far-Western experiments were carried out by first incubating recombinant DNMT1 with equimolar amounts of either recombinant SET7 or MBP2 (New England Biolabs), as described previously (28). UNC1215 and proteasome inhibitor MG132 were purchased from Tocris Bioscience and Selleck Chemicals, respectively. UNC1215 and MG132 were dissolved in ethanol and methanol, respectively. For cell cycle analysis, HeLa cells were synchronized in G_1_/S by using 2 mm thymidine block followed by 5 μg/ml aphidicolin treatment. Chromatin was extracted as described previously (12). For DNMT1 protein stability and degradation studies, either cycloheximide or MG132 was applied. For the cycloheximide study, HeLa cells were treated with 60 μm UNC1215 for 2 days and then incubated with 50 μg/ml cycloheximide (Sigma-Aldrich) at different times. For the MG132 study, HeLa cells were treated with different concentrations (0, 20, 40, and 80 μm) of UNC1215 for 2 days and then incubated with 50 μm MG132 for 2 h. Cells were then lysed and assayed by Western blot using a DNMT1 antibody (New England Biolabs, catalog no. M0231S).

Immunoprecipitations of nuclear cell extracts (400 μg to 1 mg) were performed by using 5 μg of anti-DNMT1 (Abcam, catalog no. ab92453) or anti-PHF20L1 (Sigma-Aldrich, catalog no. HPA028417). 5 μg of purified normal rabbit IgG (Cell Signaling Technology, catalog no. 2729) was used as a negative control. Immunoprecipitations were loaded into SDS-polyacrylamide gels for subsequent Western blot analyses. For immunoprecipitation studies done after UNC1215 treatment, HeLa cells were treated with 80 μm UNC1215 or ethanol for 3 days prior to precipitation.

GST pull-downs were performed with increasing amounts (1, 2, and 4 μg) of baculovirus purified full-length DNMT1 (New England Biolabs, catalog no. M0230S) that were methylated overnight by MBP-SET7 (New England Biolabs, catalog no. M0233S) and then incubated with purified MBT-GST (10 μg). DNMT1 was cloned into pVIC1 (New England Biolabs) and purified as described previously (32). MBP-SET7 and MBP-PHF20L1 were cloned into pMAL-C5x vector (New England Biolabs, catalog no. N8108S) and purified using amylose resin (New England Biolabs, catalog no. E8021).

*In vitro* pull-downs of MBP-PHF20L1a with DNMT1K142me1 peptide were carried out by first incubating 10 μm peptide (biotin-LSKPRTPRRSK(me1)SDGEAKPE) with agarose-streptavidin beads (25 μl of bead slurry, Thermo Scientific, catalog no. 2359) for 1 h at 4 °C with rotation in peptide binding buffer containing 10% glycerol (v/v), 0.1 mm DTT, 1 mm EDTA, 20 mm HEPES, and 100 mm KCl. The beads were then washed two times with 1× PBS and resuspended in fresh binding buffer. 500 ng of recombinant MBP-PHF20L1a, with increasing concentrations of UNC1215 inhibitor (0, 20, 40, 100, and 200 μm) was then added simultaneously to the peptide-bound streptavidin beads and incubated for 1 h at 4 °C with rotation. The beads were then washed two times with binding buffer and resuspended in 3× SDS sample buffer containing 1 mm DTT (New England Biolabs, catalog no. B7709S). Samples were then boiled at 95 °C for 5 min and analyzed by Western blot analysis (detailed under “Western Blot and Densitometry”) with anti-MBP-HRP antibodies at a 1:5000 dilution (New England Biolabs, catalog no. E8038S).

For immunofluorescence studies, COS-7 cells were cultured on coverslips and transfected with a mixture of epitope-tagged PHF20L1a, CFP-SET7 plasmids, and Transpass D2 transfection reagent (New England Biolabs) at a ratio of 1:3 μg/μl for 24 h. The cells were fixed with 1% paraformaldehyde (v/v). Serum detecting endogenous DNMT1K142me1 protein (New England Biolabs) was used at a 1:10,000 dilution and visualized with an anti-rabbit IgG coupled with Alexa Fluor 594 dye (Molecular Probes). Epitope-tagged PHF20L1a protein was detected by an anti-mouse FLAG (or anti-mouse Myc from Cell Signaling Technology, catalog no. 2276) and visualized with an anti-mouse IgG coupled with Alexa Fluor 488 dye (Molecular Probes). These probes were visualized under a Zeiss LSM510 confocal microscope with a 63× oil objective lens or under a Zeiss Axiovert 200M microscope. CFP-SET7 was visualized using confocal microscopy (405-nm laser) or with an AQUA filter using an Axiovert microscope. In some cases, DAPI nuclear staining was used. For 5mC detection, fixed cells were first incubated with 2 n HCl for 20 min and neutralized with 1 m Tris base for 5 min at room temperature. A mouse monoclonal antibody specific for 5mC (Eurogentec, catalog no. BI-MECY-0100) was added at 1:500 dilution overnight at 4 °C and detected by an anti-mouse IgG coupled with Alexa Fluor 488 dye. Endogenous PHF20L1 and DNMT1 proteins in HeLa cells were detected using an anti-PHF20L1 antibody (Abcam, catalog no. ab67796) at 1:20 dilution and an anti-DNMT1 antibody (Santa Cruz Biotechnology, Inc., catalog no. sc-20701) at 1:50 dilution. To visualize endogenous colocalization of PHF20L1 and DNMT1, secondary anti-mouse IgG coupled with Alexa Fluor 594 dye and anti-rabbit IgG coupled with Alexa Fluor 488 dye antibodies (Molecular Probes) were used, respectively.

For the far-Western experiment, samples containing recombinant DNMT1 (10 μg) were first incubated with either recombinant MBP2-SET7 (40 μg) or MBP2 (20 μg). Increasing amounts of these DNMT1 mixtures (0.25, 0.5, 1, and 2 μg) were then electrophoresed on an SDS-polyacrylamide gel and transferred to a PVDF membrane. The PVDF membrane was blocked in 1× PBS, 0.1% Tween 20 (v/v), and 5% dry milk (w/v) for 1 h at room temperature. The membrane was then washed three times for 5 min in 1× PBS containing 0.1% Tween 20 (v/v). Peptide binding buffer containing 2 μg of recombinant MBP-PHF20L1a was added to the membrane and incubated for 1 h at room temperature. The membrane was then washed three times for 5 min in 1× PBS, 0.1% Tween 20 (v/v). Rabbit anti-PHF20L1 (Sigma-Aldrich, catalog no. HPA028417) antibody was used (1:2000 dilution) to detect the PHF20L1a bound to the membrane-immobilized DNMT1.

##### siRNA Knockdown of PHF20L1

For *PHF20L1* gene knockdown, HeLa cells were transfected for 48 h using HiPerFect reagent (Qiagen, catalog no. 301705) with 100 nm PHF20L1 siRNA (catalog no. EHU060221, Sigma-Aldrich) or SMART pool ON-TARGETplus PHF20L1 siRNA (catalog no. L-027322-01-0005, Thermo Scientific). For control transfection, 100 nm EGFP siRNA (catalog no. EHUEGFP, Sigma-Aldrich) or a universal negative control siRNA were used (catalog no. 12935-300, Invitrogen). The siRNA control used for SMART pool PHF20L1 was an ON-TARGETplus non-targeting control pool (catalog no. D-001810-10-05, Thermo Scientific). For global 5mC detection by HPLC, cells were transfected for 5 days using 50 nm PHF20L1 siRNA.

##### Subcellular Biochemical Fractionation

Briefly, 10^7^ HeLa cells were collected, washed three times with PBS, and resuspended in buffer A containing 10 mm HEPES (pH 7.4), 1.5 mm MgCl_2_, 10 mm KCl, 0.34 m sucrose, 10% glycerol (v/v), 0.1% Triton X-100 (v/v), 1 mm DTT, protease inhibitor mixture (Sigma-Aldrich), and PMSF. After a 5-min incubation on ice, cells were centrifuged (1300 × *g* for 5 min at 4 °C), and the pellet P1 corresponding to the nuclei was collected, the supernatant S1 representing the cytoplasmic fraction. The nuclei were lysed for 10 min on ice with a hypotonic buffer B containing 0.2 mm EGTA, 3 mm EDTA, 1 mm DTT, and protease inhibitor mixture plus PMSF. The soluble nuclear fraction S3 and the chromatin P3 were separated through centrifugation (1700 × *g* for 5 min at 4 °C). The P3 fraction was resuspended in buffer C, containing 10 mm Tris-HCl (pH 7.5), 1 mm CaCl_2_, and protease inhibitor mixture plus PMSF. The chromatin P3 fraction was kept at −80 °C. Before loading 20 μg of chromatin on a SDS-polyacrylamide gel, the P3 fraction was first treated with DNase I (New England Biolabs, catalog no. M0303S) for 10 min at 37 °C and then resuspended and boiled for 5 min in SDS sample buffer (New England Biolabs) containing 1 mm DTT.

##### Gel Filtration Chromatography

Gel filtration chromatography was performed with Jurkat cell nuclear extract as described previously (30).

##### In Vivo Ubiquitination Assay

COS-7 cells were co-transfected with a mixture of GFP or GFP-SET7, 3xFLAG or 3xFLAG-PHF20L1, HA-ubiquitin as well as DsRed-DNMT1 plasmids, and Transpass D2 transfection reagent (New England Biolabs) at a ratio of 1:3 μg/μl for 24 h. Transfected COS-7 cells were treated with 10 μm MG132 for 12 h and lysed as described previously (27). Cell lysates (100–200 μg) were then immunoprecipitated with DNMT1 antibody (Abcam, catalog no. ab92453). Ubiquitination was detected as described previously (27).

##### Western Blot and Densitometry

Western blots were performed as previously described (30). Antibodies and antiserum against DNMT1 were obtained from New England Biolabs (1:10,000 working dilution). Anti-PHF20L1 antibody (1:5000 working dilution) was purchased from Sigma-Aldrich (catalog no. HPA028417). Anti-SET7 (catalog no. 2813), anti-histone H3 (catalog no. 9715), anti-PHF20 (catalog no. 3934), and anti-HA tag (catalog no. 3724) were obtained from Cell Signaling Technology and used at a 1:2000 dilution. Anti-β-actin and anti-GFP monoclonal antibodies were obtained from Sigma-Aldrich and Roche Applied Science, respectively, and used at a 1:5000 dilution. Densitometry was performed using ImageJ software (W. S. Rasband, National Institutes of Health). All densitometry values (arbitrary units) were normalized to either their respective β-actin, histone H3 blots, or Ponceau S staining.

##### Quantitative PCR Analysis of PHF20L1 siRNA Knockdowns

siRNA-transfected HeLa cells were harvested, and total RNA was purified using TRIzol reagent (Invitrogen). For reverse transcription of RNA, iScript (Bio-Rad) was used to convert 1 μg of total RNA, and then 40 ng of cDNA was quantified by performing real-time PCR using a MyiQ Cycler (Bio-Rad) and iQ SYBR Green Supermix (Bio-Rad). The following primers were used in the real-time mixes at a working concentration of 5 nm each: DNMT1, GGCTGAGATGAGGCAAAAAG (forward) and ACCAACTCGGTACAGGATGC (reverse); PHF20L1, AGCGTTGGAACCATCGTTAT (forward) and GACAATCAGACCAGCAAGCA (reverse). RNA values were normalized using primers GCCAAAAGGGTCATCATCTC (forward) and TGAGTCCTTCCACGATACCA (reverse) for human GAPDH.

##### Nucleoside Digestion and LC-MS Analysis

Genomic DNA was isolated from siRNA-transfected HeLa cells using an Invitrogen Easy-DNA kit (catalog no. K1800-01) per the manufacturer's specifications. RNA contamination was removed from the genomic DNA with AMPure XP (Agencourt, catalog no. A63880) beads per the manufacturer's specifications. Nucleoside digestion of genomic DNA was done by incubating 4–5 μg of genomic DNA in a mixture of New England Biolabs nucleases at 37 °C overnight. Nucleosides were subsequently purified by running the digested sample through a Qiagen QIAquick spin column and collecting the flow-through. LC-MS analysis was performed on an Agilent 1200 series HPLC system equipped with a G1316A UV detector and 6120 mass detector (Agilent, Santa Clara, CA) with a Waters Atlantis T3 column (4.6 × 150 mm, 3 μm, Waters (Milford, MA)) equipped with an in-line filter and guard column. Peak quantification was based on the integration area of each target nucleoside at the maximum absorption of UV and adjusted by its respective extinction coefficient constant.

##### CADOR Peptide Arrays

Biotin or biotin-DNMT1K142me1 peptides were bound and detected on a chromatin-associated domain array (CADOR) as described previously (24). The CADOR chip contains bromodomain, chromodomain, and Tudor, PHD, SANT, SWIRM, MBT, CW, and PWWP domains fused to glutathione *S*-transferase (GST).

## RESULTS

### 

#### 

##### MBT Domain of PHF20L1 Binds Methylated DNMT1

**FIGURE 1.**
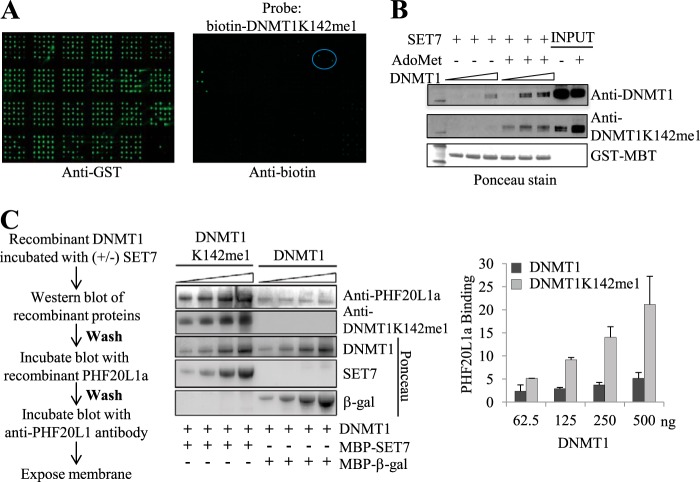
**PHF20L1a binds to DNMT1K142me1 *in vitro*.** *A*, binding of DNMT1K142me1 peptide to a CADOR. Anti-GST antibody illustrates the bromodomain, chromodomain, and Tudor, PHD, SANT, SWIRM, MBT, CW, and PWWP domains fused to GST and their spotting on the array. Binding of biotin-tagged DNMT1K142me1 peptide to the CADOR revealed the MBT domain of PHF20L1 as a binding partner, shown in a *circle*. The *four spots* observed in the PHD-containing block on the *left* represent positive controls. *B*, recombinant DNMT1, methylated by SET7 at Lys-142, binds to the MBT domain of PHF20L1. The DNMT1, SET7, and *S*-adenosylmethionine (*AdoMet*) reaction composition is indicated at the *top* of the blot. Ponceau staining of transferred proteins from the MBT pull-down are shown along with Western blot using anti-DNMT1 and anti-DNMT1K142me1 antibodies. *C*, far-Western blot revealing recombinant PHF20L1a binding to recombinant full-length DNMT1K142me1. A schematic flow diagram of the far-Western blot is detailed to the *left*. Reaction conditions for the loaded proteins are indicated at the *bottom* of the blot. Ponceau staining of transferred proteins from the reactions is shown along with the far-Western antibody, anti-PHF20L1a, and the Western blot antibody, anti-DNMT1K142me1. Densitometry measurements of either DNMT1 (*black*) or DNMT1K142me1 (*gray*) binding to PHF20L1a are shown to the *right* of the far-Western blot (arbitrary units). *Error bars*, S.D.

**FIGURE 2.**
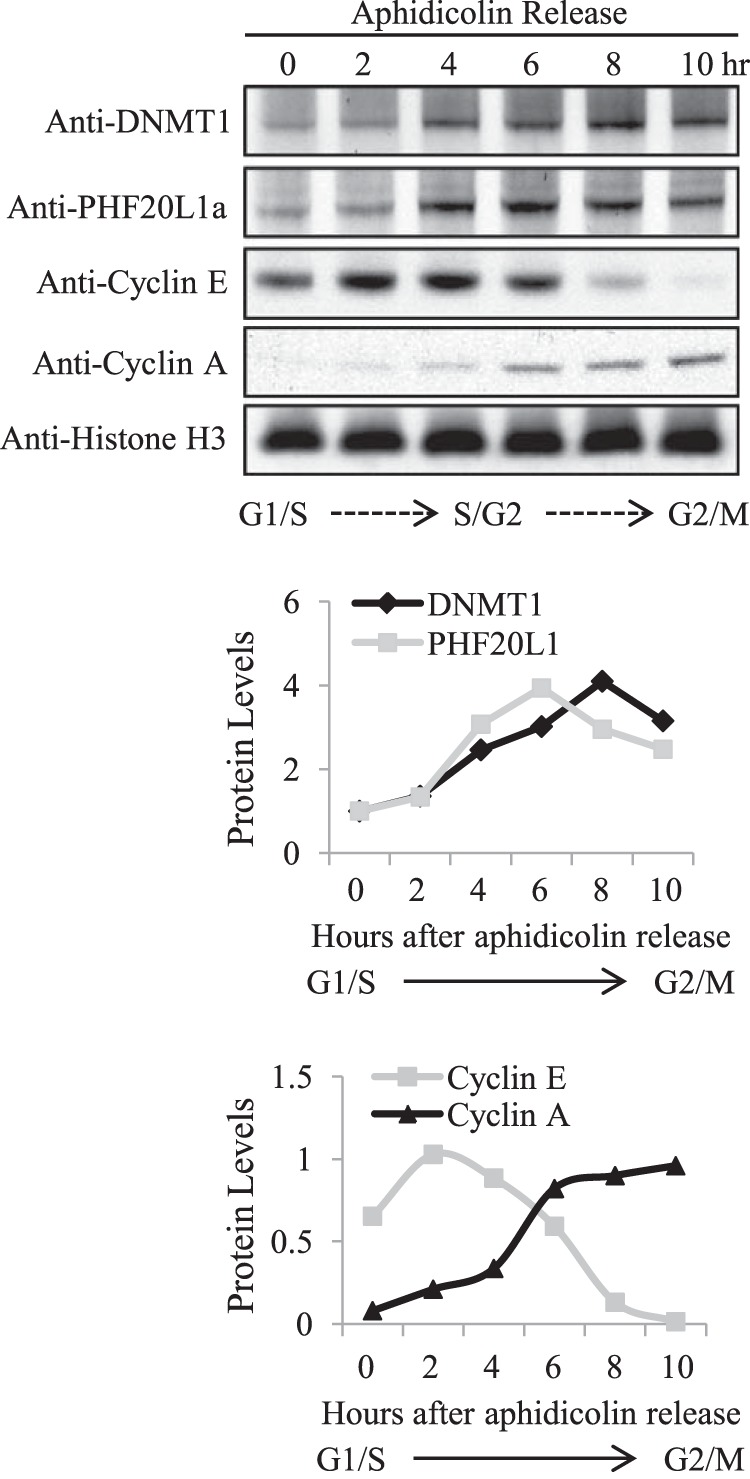
**Expression of DNMT1 and PHF20L1a coincide during cell cycle progression.** HeLa cells were first treated with thymidine and subsequently aphidicolin for late G_1_ arrest. Aphidicolin was then removed, allowing for cell cycle release (hours after release indicated at the *top*, and corresponding cell cycle stages indicated at the *bottom*). Chromatin-extracted samples were analyzed by Western blot analysis with the indicated antibodies. Densitometry measurements for protein levels of DNMT1, PHF20L1a, and cell cycle progression markers cyclin E and cyclin A, normalized to histone H3, were plotted *below* (arbitrary units).

To search for a reader of DNMT1K142me1 modification, we synthesized a biotin-tagged peptide encompassing amino acids 132–150 of DNMT1 with monomethylated Lys-142 in the center and screened with a protein domain microarray (24, 34) to identify potential readers. Indeed, the MBT domain of PHF20L1 was specifically bound to the methylated DNMT1 peptide (Fig. 1*A*). To validate if the MBT domain of PHF20L1 is a true DNMT1K142me1 reader, GST pull-down experiments were performed using increasing amounts of purified full-length recombinant DNMT1 incubated with constant amounts of SET7 lysine methyltransferase, *S*-adenosylmethionine methyl donor, and the GST-MBT domain fusion derived from PHF20L1. Upon Lys-142 methylation of recombinant DNMT1 by SET7, GST-MBT domain bound more robustly, compared with control unmethylated DNMT1 (Fig. 1*B*). To confirm that this interaction takes place between full-length proteins, we methylated DNMT1 with SET7, separated both mock-methylated (unmethylated) and methylated DNMT1 by SDS-PAGE, transferred the proteins to a membrane, and then incubated the blot with MBP-PHF20L1 full-length fusion protein as described schematically (Fig. 1*C*, *left*), similar to far-Western blotting. As expected, full-length PHF20L1 was bound to DNMT1K142me1 in a dose-dependent manner, and the interaction was virtually abolished without SET7 methylation in the mock-methylated DNMT1 (Fig. 1*C*, *middle*). We observed between 2- and 3-fold more binding between full-length PHF20L1 and DNMT142me1 as compared with control (Fig. 1*C*, *right*). These observations led us to believe that both methylated DNMT1 and PHF20L1 may form a complex in mammalian cells. Because DNMT1 is a maintenance methyltransferase and is required during S phase of the cell division, we monitored the expression of both PHF20L1 and DNMT1 in a synchronized population of cells by Western blot. Both proteins are expressed in a cell cycle-dependent manner with total DNMT1 protein level peaking at 8 h and PHF20L1 at 6 h after aphidicolin release (Fig. 2). This suggests that during early stages of DNA synthesis, there is an abundance of PHF20L1, allowing it to form a complex with methylated DNMT1.

##### PHF20L1 Interacts with DNMT1 in Mammalian Cells

**FIGURE 3.**
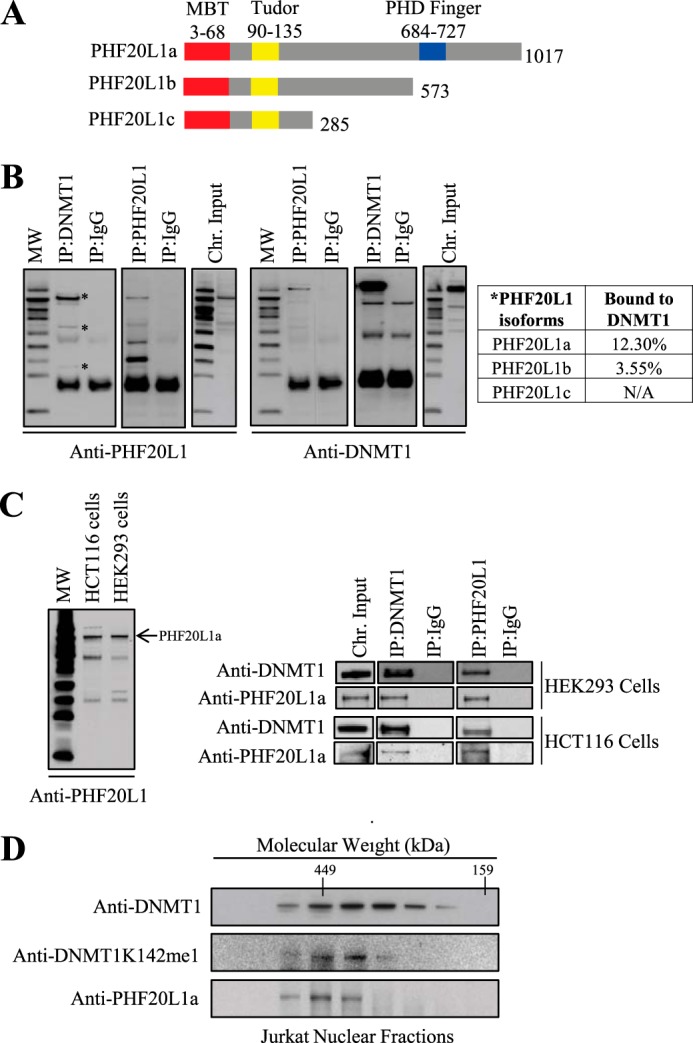
**PHF20L1 binds to DNMT1 *in vivo*.** *A*, schematic illustrating the three different known isoforms of PHF20L1. All three isoforms contain an MBT and Tudor domain, but only isoform a, the largest, contains a PHD finger domain. *B*, co-immunoprecipitation (*IP*) of DNMT1 and PHF20L1 reveals *in vivo* binding of these two proteins. Molecular weight marker and immunoprecipitation antibodies, anti-DNMT1, anti-PHF20L1, and anti-IgG, are indicated at the *top* of the Western blots. Antibodies used for protein detection, anti-PHF20L1 and anti-DNMT1, are indicated to the *bottom* of the Western blots. Immunoprecipitations revealed that DNMT1 binds all three isoforms of PHF20L1 (indicated by an *asterisk*), but the largest PHF20L1a isoform is the predominant species bound to DNMT1 on the chromatin. Densitometry measurements of PHF20L1 immunoprecipitated with DNMT1 antibody were compared with its corresponding chromatin (*chr*.) input sample to determine the percentage of PHF20L1 bound to DNMT1. *C*, PHF20L1a binds DNMT1 in human HEK293 and HCT116 cells. On the *left* is a Western blot revealing that PHF20L1a (indicated by an *arrow*) is the predominant chromatin binder in both HEK293 and HCT116 cells. To the *right* are Western blots of DNMT1 and PHF20L1a co-immunoprecipitations in HEK293 and HCT116 cells, revealing *in vivo* interaction of these two proteins. *D*, size exclusion chromatography fraction of nuclear extract, demonstrating co-elution of DNMT1K142me1 and PHF20L1a with molecular weight markers indicated at the *top*. Western blot antibodies for DNMT1, DNMT1K142me1, and PHF20L1a are shown to the *left*.

**FIGURE 4.**
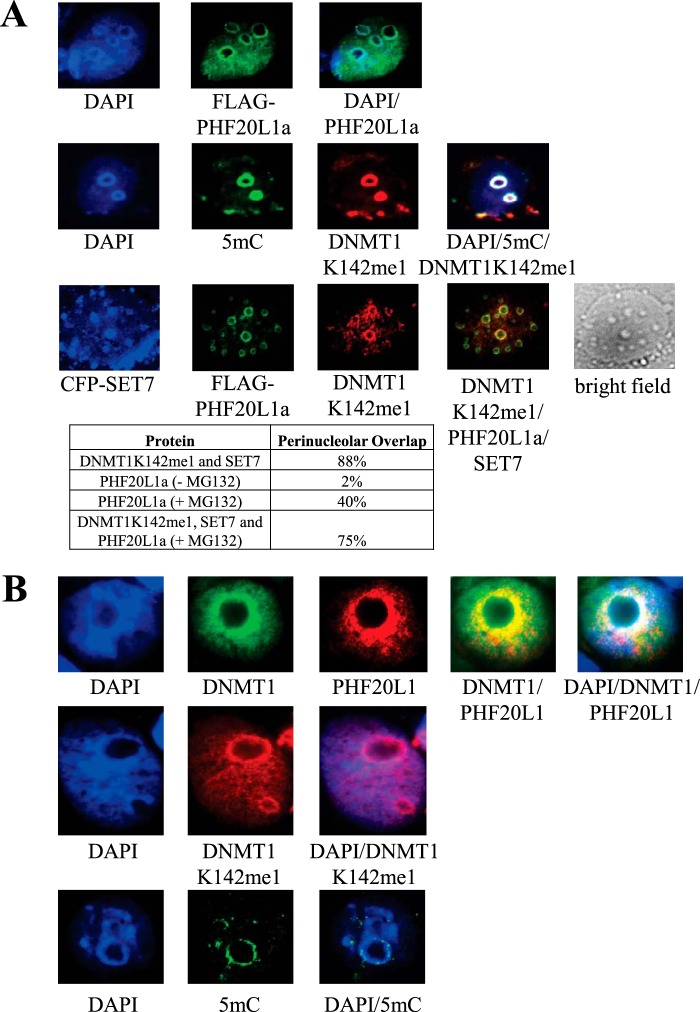
**Cellular location and colocalization of PHF20L1a.** *A*, the *top panel* shows pericentric localization of PHF20L1a. DNA staining is DAPI. In the *middle panel*, 5mC is shown in *green* and DNMT1K142me1 in *red*. In the *bottom panel* are shown CFP-SET7 in *blue* along with PHF20L1a in *green* and DNMT1K142me1 in *red*. The *merge panel* of DNMT1K142me1, PHF20L1a, and SET7 shows circular pericentric heterochromatin matching to the bright field image. Percentage perinucleolar localization was calculated from 200 transfected cells. *B*, colocalization of endogenous DNMT1, PHF20L1, and 5-methylcytosine in pericentric heterochromatin in HeLa cells. DAPI staining shows the heterochromatic regions.

Western blot analysis revealed that HeLa cells are a good source for both DNMT1 and PHF20L1, and DNMT1 expression is cell cycle-regulated, as expected (Fig. 2). PHF20L1 has three isoforms, PHF20L1a, -b, and -c, and they all share the same MBT binding domain. PHF20L1a is the largest isoform and it is the only one containing a PHD (Fig. 3*A*). The PHD has been shown to bind methylated lysine tails on histones and to participate in mammalian gene expression (35). Co-immunoprecipitation using anti-DNMT1 antibody predominantly pulled out the PHF20L1a isoform. In a reciprocal immunoprecipitation using anti-PHF20L1 antibody, we pulled out DNMT1 from the nuclear extract of HeLa cells, thus demonstrating complex formation between both DNMT1 and PHF20L1a on the chromatin (Fig. 3*B*). Furthermore, PHF20L1a was the predominant isoform bound to chromatin in HCT116 and HEK293 cells (Fig. 3*C*, *left*), and this isoform was also confirmed bound to endogenous DNMT1 in these cells (Fig. 3*C*, *right*). Due to the predominance of PHF20L1a across three different mammalian cell types and its binding to endogenous DNMT1, we have focused our subsequent studies only on the PHF20L1a isoform when examining its interaction with DNMT1 in the cell. PHF20L1a acting as a binding partner of DNMT1 was also confirmed by Western blot of nuclear extract fractions from a gel filtration column, which revealed that DNMT1K142me1 co-eluted with the PHF20L1a isoform (Fig. 3*D*). We also performed a series of expression studies to investigate the colocalization of DNMT1 and PHF20L1a. A typical colocalization between endogenous DNMT1K142me1 and epitope-tagged PHF20L1 (3xFLAG-PHF20L1/FLAG-PHF20L1a) was perinucleolar (Fig. 4*A*, *top*); similarly, 5mC and DNMTK142me1 were also perinucleolar (Fig. 4*A*, *middle*). Indeed, overexpressed SET7, epitope-tagged PHF20L1a, and DNMT1K142me1 colocalized together (Fig. 4*A*, *bottom*). In control studies, overexpression of SET7 and DNMT1K142me1 resulted in over 85% of the cells showing colocalization, as was expected and observed before (27). Overexpression of epitope-tagged PHF20L1a alone resulted in a low percentage (∼2%) of perinucleolar localization (Fig. 4*A*, *top*), and this increased to ∼40% in the presence of proteasomal inhibitor MG132. The colocalization percentage between PHF20L1 and DNMT1K142me1 increased to 75% when epitope-tagged PHF20L1a was overexpressed in the presence of MG132 inhibitor, suggesting that more cellular PHF20L1a aids in protection of DNMT1K142me1 (Fig. 4*A*, *bottom table*). The colocalization remained perinucleolar after staining the cells with either anti-FLAG (N-terminal tag of PHF20L1a) or anti-Myc (C-terminal tag of PHF20L1a) antibody, demonstrating that it is indeed the PHF20L1a isoform that is in the perinucleolar complex (data not shown). Therefore, blocking proteasomal degradation of DNMT1K142me1 resulted in the stabilization and capture of DNMT1K142me1-PHF20L1 complexes in the cells. Furthermore, immunostaining of endogenous DNMT1 and PHF20L1 showed perinucleolar localization in HeLa cells, as was observed with the tagged fusion constructs (Fig. 4*B*, *top*). Similarly, DNMT1K142me1 and 5mC are also present in the perinucleolar region in untransfected HeLa cells, as expected (Fig. 4*B*, *middle* and *bottom*).

##### Chromatin Loading of DNMT1 Is Facilitated by PHF20L1

**FIGURE 5.**
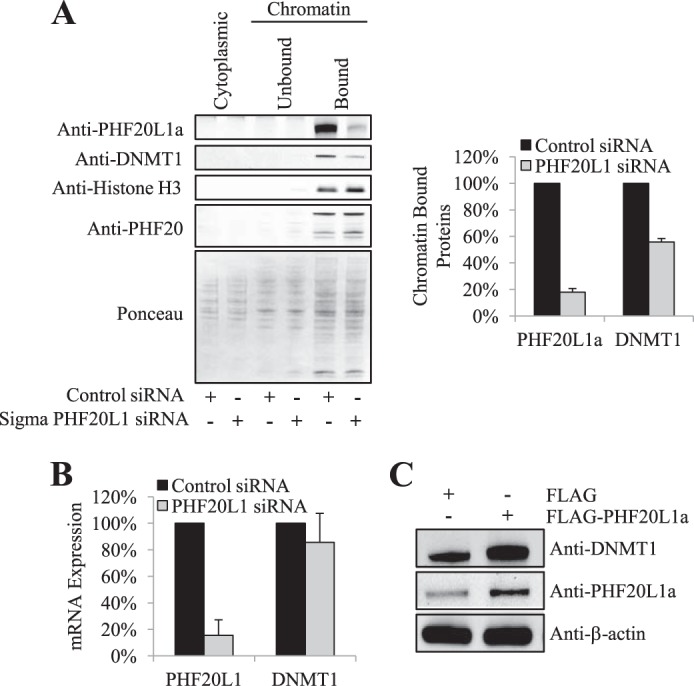
**Chromatin-bound DNMT1 stability is facilitated by PHF20L1.** *A*, chromatin-bound DNMT1 decreases in response to PHF20L1 knockdown. Cytoplasmic, unbound, and bound chromatin fractions are indicated at the *top*, whereas the control and PHF20L1 siRNA transfections are indicated at the *bottom* of the Western blot. Equal loading of whole cell extracts was revealed by Ponceau staining. Anti-PHF20L1a, anti-DNMT1, anti-histone H3, and anti-PHF20 antibodies were used for protein detection in the Western blot analysis. Densitometry measurements for chromatin-bound PHF20L1a and DNMT1, normalized to total protein levels, after either control (*black*) or PHF20L1 (*gray*) siRNA transfections are shown to the *right* (arbitrary units). *B*, knockdown of PHF20L1 has minimal effect on the expression of DNMT1 mRNA. Shown is a graph detailing the percentage of PHF20L1 (primer measures all isoforms) and DNMT1 mRNA expression after either control (*black*) or PHF20L1 (*gray*) siRNA transfection. *C*, overexpression of 3xFLAG-tagged PHF20L1a (FLAG-PHF20L1a) increases global levels of DNMT1. Overexpression of either FLAG or FLAG-PHF20L1a is indicated at the *top*, whereas Western blot antibodies are indicated to the *right* of the Western blot. *Error bars*, S.D.

PHF20L1a contains two more structural domains apart from the MBT domain. Both Tudor and PHD domains are readers of lysine methylation on histone H4 and H3, respectively. Similarly, DNMT1 also loads itself onto chromatin during cell division. Therefore, we investigated if PHF20L1 participates in epigenetic inheritance mechanisms by interacting with DNMT1. We knocked down PHF20L1 using a synthetic siRNA and isolated cytoplasmic and nuclear fractions. The nuclear fractions were further processed to obtain chromatin-bound and -unbound fractions. Cytoplasmic, chromatin-unbound, and chromatin-bound protein fractions were Western blotted and probed for PHF20L1a. Both PHF20L1a and DNMT1 were found to be exclusively chromatin-bound (Fig. 5*A*, *left*). siRNA knockdown of PHF20L1 resulted in reduction of chromatin-bound DNMT1 (Fig. 5*A*, *left* and *right*). After a single round of siRNA transfection, PHF20L1 mRNA expression was down-regulated over 80% (Fig. 5*B*). Also, an additional PHF20L1 siRNA was utilized from a different manufacturer, confirming these findings (data not shown). Coinciding with our siRNA knockdown finding, overexpression of FLAG-PHF20L1a increased the levels of DNMT1 protein when compared with FLAG alone (Fig. 5*C*). These findings suggest that PHF20L1 prevents the proteasomal degradation of DNMT1, thus increasing DNMT1 levels on chromatin.

##### PHF20L1 Masks Lysine-methylated DNMT1 from Proteasomal Degradation

**FIGURE 6.**
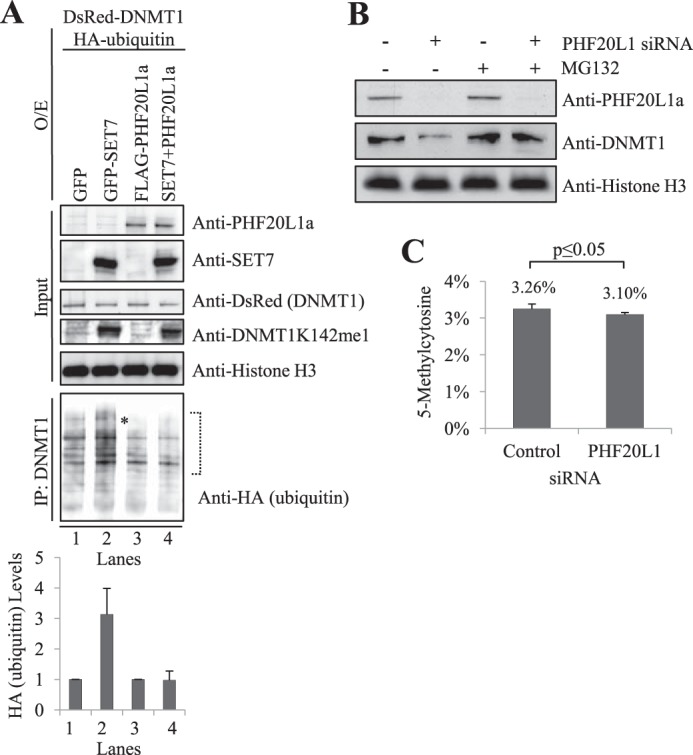
**PHF20L1a inhibits DNMT1K142me1 ubiquitination and degradation.** *A*, FLAG-PHF20L1a overexpression prevents ubiquitination of DNMT1K142me1. Overexpression (*O/E*) of GFP, GFP-SET7, FLAG-PHF20L1a, or a combination of GFP-SET7 and FLAG-PHF20L1a are indicated at the *top* of the Western blot. Overexpression of DsRed-DNMT1 and HA-ubiquitin and subsequent treatment with the MG132 proteasome inhibitor were carried out for all samples. Immunoprecipitations (*IP*) with anti-DNMT1 antibody were subsequently performed, and the precipitate was analyzed by Western blot. Input was revealed with anti-PHF20L1a, anti-SET7, anti-DsRed-DNMT1, anti-DNMT1K142me1, and anti-histone H3 antibodies, whereas DNMT1 immunoprecipitations were revealed with anti-HA-ubiquitin antibody (indicated on the *right*). Full-length HA-ubiquitinated DNMT1 was present (*), but smaller degradation products were prevalent. Densitometry measurements of anti-HA-ubiquitin, normalized to histone H3, were done within the indicated *brackets* to show levels of ubiquitinated full-length DNMT1 (arbitrary units). *B*, loss of chromatin-bound DNMT1 in response to PHF20L1 knockdown is mediated by proteasomal degradation. siRNA-mediated knockdown of PHF20L1 and subsequent treatment with the MG132 proteasomal inhibitor are indicated at the *top* of the Western blot. Anti-PHF20L1a, anti-DNMT1, and anti-histone H3 antibodies used in the Western blot are indicated to the *right* of the blot. *C*, LC-MS analysis reveals that siRNA-mediated knockdown of PHF20L1 significantly reduces global levels of 5-methylcytosine. Genomic DNA was digested to single nucleosides for LC-MS analysis. Methylation changes were normalized to total levels of corresponding nucleotides. Total levels of 5-methylcytosine were significantly lower (*p* = 0.029) after transfection of PH20L1 siRNA compared with control siRNA. Data represent two experimental replicates and four technical replicates. *Error bars*, S.D.

Because DNMT1 degradation relies on Lys-142 monomethylation and DNMT1K142me1 binds to PHF20L1, we next examined whether ubiquitin-mediated degradation is targeting this complex. Previous studies have established that DNMT1 degradation is mediated by the ubiquitin-proteasome pathway (19). Because the majority of cellular proteins destined for degradation are polyubiquitinated (36), we also examined whether polyubiquitination of DsRed-DNMT1 fusion is preventable by PHF20L1. Expression constructs containing isoform PHF20L1a and HA-ubiquitin were transfected into the cells, with or without GFP-SET7, in the presence of proteasome inhibitor MG132, and DNMT1 was subsequently immunoprecipitated. We performed Western blot analysis on the immunoprecipitated materials with an anti-HA antibody (Fig. 6*A*). In the presence of SET7 (Fig. 6*A*, *lane 1 versus lane 2*), immunoprecipitated DNMT1 showed a stronger smear of HA-ubiquitin-tagged DNMT1, confirming our previously reported observation that methylated DNMT1 is the target for proteasomal degradation (27). However, overexpression of PHF20L1a, along with SET7, resulted in a reduction of the HA-ubiquitin-tagged smear (Fig. 6*A*, *lane 2 versus lane 4*). Densitometry analysis of the HA-ubiquitin smear quantitatively revealed that ubiquitinated DNMT1 was at least 3-fold higher in the presence of SET7 but was reduced to background levels when PHF20L1a and SET7 were expressed together (Fig. 6*A*, *bottom*). In the absence of exogenous SET7, the ubiquitinated DNMT1 level did not change despite overexpression of PHF20L1a (Fig. 6*A*, *lane 1 versus lane 3*). Overall, these observations suggest that in the presence of PHF20L1a, lysine ubiquitination of DNMT1 occurs at or below background levels. Further validating this finding, siRNA knockdown of PHF20L1a in the presence of the MG132 proteasome inhibitor prevented DNMT1 degradation on chromatin, whereas siRNA knockdown in cells not treated with MG132 showed a 70% loss of DNMT1 (Fig. 6*B*). Overall, these results strongly suggest that DNMT1K142me1 degradation by the ubiquitin-proteasome pathway is at least partly prevented by PHF20L1a masking of the monomethylated Lys-142 on DNMT1.

##### Depletion of PHF20L1 Leads to DNA Methylation Changes

Because the MBT domain of PHF20L1 binds methylated DNMT1, protecting it from SET7-mediated degradation, it is plausible that PHF20L1 may participate in DNA methylation of the genome. To test our hypothesis, we depleted PHF20L1 using siRNA and measured the levels of 5-methylcytosine in the genome. We observed a subtle (∼5%) but significant (*p* = 0.029) hypomethylation of the genome, suggesting that changes in DNA methylation occur in the absence of PHF20L1 (Fig. 6*C*). The hypomethylation change that was observed upon PHF20L1 siRNA knockdown coincides with our previous findings in which SET7 was overexpressed, leading to a 6.8% hypomethylation of genomic DNA (27). The similarity between these findings provides further evidence that PHF20L1 and SET7 are direct antagonists in the regulation of DNMT1 proteasomal degradation.

##### UNC1215 Blocks DNMT1 MBT Domain Interaction

**FIGURE 7.**
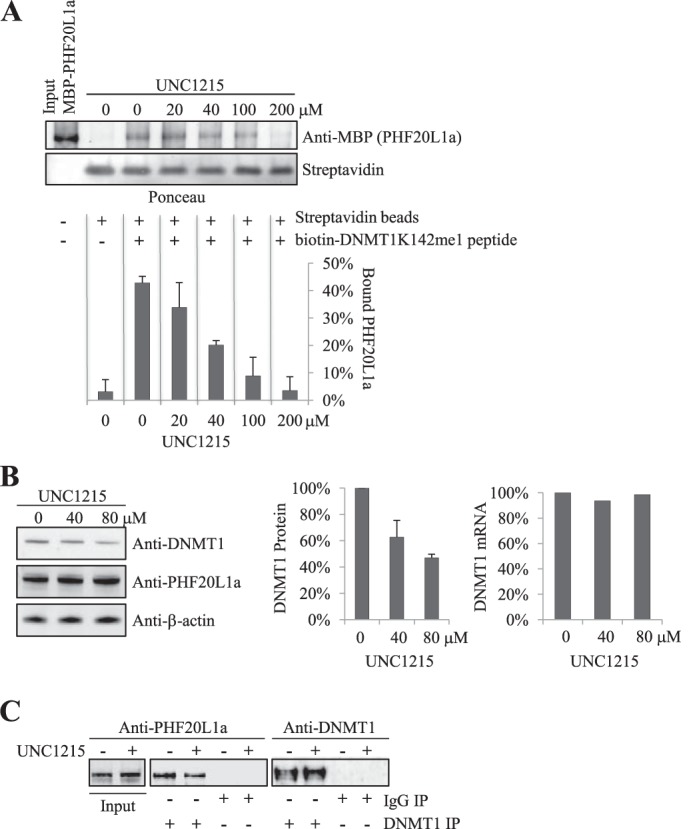
**UNC1215 blocks DNMT1K142me1 MBT domain interaction.** *A*, UNC1215 inhibits the binding of biotin-DNMT1K142me1 peptide to recombinant PHF20L1a *in vitro*. Constant amounts of recombinant MBP-PHF20L1a and biotin-tagged DNMT1K142me1 peptide prebound to streptavidin beads with increasing concentrations of UNC1215 are indicated at the *top*. Equal loading of streptavidin was illustrated by Ponceau staining, whereas anti-MBP antibody was used to reveal MBP-PHF20L1a. Densitometry measurements of MBP-PHF20L1a, normalized to streptavidin, are shown at the *bottom* and illustrate the percentage of bound MBP-PHF20L1. *B*, UNC1215 treatment decreases levels of DNMT1 in HeLa cells. Cells were treated with increasing concentrations of UNC1215 (indicated at the top), and extracts were analyzed by Western blot (antibodies indicated to the *right*). Densitometry measurements illustrating the percentage of DNMT1 protein levels, normalized to β-actin, are shown in the *middle panel*. Real-time quantitative PCR measurement of DNMT1 mRNA in response to UNC1215 treatment is shown in the *far right panel. C*, UNC1215 treatment of HeLa cells reduces *in vivo* binding of PHF20L1 to DNMT1. DNMT1 immunoprecipitations (*IP*) were performed on HeLa cells treated with either 80 μm UNC1215 (+) or ethanol (−). Western blot antibodies for DNMT1 or PHF20L1 were then used to visualize the DNMT1 immunoprecipitation. *Error bars*, S.D.

To unequivocally determine the functional association of MBT domain with the protection of methylated DNMT1 from degradation, we used the small molecule MBT domain inhibitor UNC1215. This cell-permeable inhibitor has been shown to be a selective chemical probe for the methyllysine-reading function of MBT domain containing protein L3MBTL3, a member of a family of chromatin-interacting transcriptional repressors (37). UNC1215 binds to the MBT domain of L3MBTL3 and competitively displaces mono- or dimethyllysine-containing peptides. Also, *in vitro* experiments have confirmed that UNC1215 interacts with the MBT domain of PHF20L1a (37). We first tested if UNC1215 treatment of PHF20L1a would prevent complex formation with DNMT1K142me1 *in vitro*. We immobilized biotinylated methylated peptide representing DNMT1K142me1 on streptavidin-agarose beads, washed unbound peptide, and then added increasing concentrations of UNC1215 and fixed amounts of MBP-PHF20L1a. The unbound proteins were then washed away, and the bound proteins were Western blotted and probed with antibody. As the concentration of UNC1215 increased, PHF20L1a binding to the DNMT1K142me1 peptide decreased, confirming that UNC1215 is a selective competitive inhibitor of the MBT domain of PHF20L1 (Fig. 7*A*).

We extended our studies to cultured cells by incubating cells with the UNC1215 inhibitor. We monitored the expression levels of DNMT1 mRNA by quantitative PCR and observed no changes in its expression profile (Fig. 7*B*, *right*). However, DNMT1 protein levels decreased in a dose-dependent manner as UNC1215 concentration increased (Fig. 7*B*, *left* and *middle*), confirming the involvement of PHF20L1 in maintaining DNMT1 levels. We also confirmed that UNC1215 can block the interaction of PHF20L1a and DNMT1 in cells. DNMT1 that was immunoprecipitated from HeLa cells treated with UNC1215 coprecipitated lower levels of PHF20L1 when compared with untreated cells (Fig. 7*C*). This illustrates that the MBT domain of PHF20L1a also binds DNMT1 *in vivo*.

**FIGURE 8.**
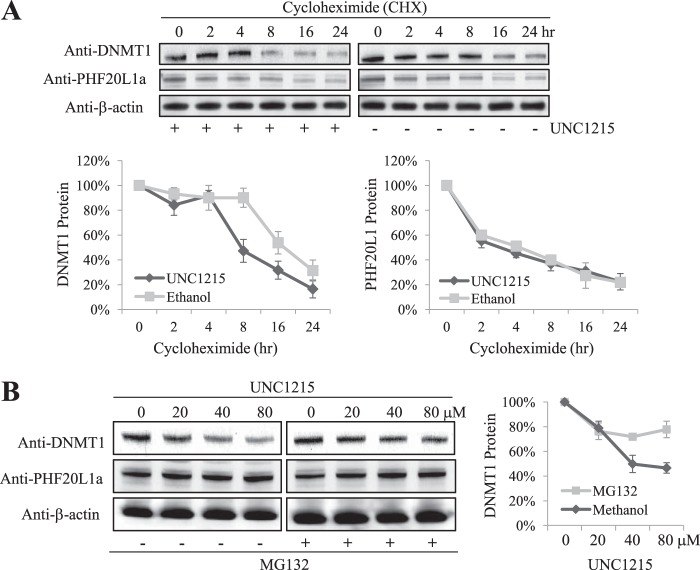
**UNC125 treatment decreases the half-life of DNMT1.** *A*, cycloheximide treatment was carried out for the indicated times shown at the *top*, after 48-h pretreatment with either 60 μm UNC1215 (+) or ethanol (−), indicated at the *bottom*. Extracted samples were analyzed by Western blotting with the indicated antibodies to the *left*. Densitometry measurements illustrating the percentage of DNMT1 and PHF20L1 protein levels, normalized to β-actin, are plotted (data are representative of three independent experiments for DNMT1 and two independent experiments for PHF20L1). *B*, proteasomal degradation mediates loss of DNMT1 after treatment with UNC1215. Cells were pretreated (48 h) with increasing concentrations of UNC1215 (indicated at the *top*) and then treated with either methanol (−) or MG132 (+) (indicated at the *bottom*), and extracts were analyzed by Western blotting with the indicated antibodies to the *left*. Densitometry measurements illustrating the percentage of DNMT1 protein levels, normalized to β-actin, are plotted to the *right* (data are representative of two independent experiments). *Error bars*, S.D.

To determine if the half-life of DNMT1 is shorter upon disruption of the PHF20L1 and DNMT1 complex, we treated HeLa cells with the protein synthesis inhibitor cycloheximide followed by either UNC1215 or vehicle control ethanol. This revealed that the half-life of DNMT1 was significantly shorter (∼50% decrease in half-life) when cells were treated with UNC1215 compared with ethanol (Fig. 8*A*, *bottom left*). UNC1215 did not have any effect on the half-life of PHF20L1 (Fig. 8*A*, *bottom right*). Coinciding with this finding, UNC1215 treatment resulted in a dose-dependent loss of DNMT1 in the control samples, which was reduced by MG132 treatment (Fig. 8*B*). Indeed, the addition of MG132 stabilized DNMT1, as was observed before (27, 38). These experiments conclusively establish that proteasomal degradation of methylated DNMT1 is regulated by the interaction of the MBT domain of PHF20L1.

## DISCUSSION

DNA methylation is a fundamental process during cell division in mammals and is responsible for maintaining genome integrity and regulating gene expression. DNMT1 adds a methyl group to hemimethylated DNA during DNA replication. After replication, the hemimethylated DNA substrate is no longer available, DNMT1 subsequently gets degraded by the ubiquitin dependent proteasome pathway, and methyllysine 142 acts as a sensor for this biological event. However, methyllysine residues are also attractive targets for methyllysine readers. Previously, only chromodomain-containing proteins were thought to be readers of methyllysine, but recently two other domains, Tudor and MBT, have been shown to bind methylated lysines on the amino terminus tails of histone H3 and H4 (39). Indeed, the MBT family of proteins can interact with core histones effectively, and this interaction can be more specific, with a higher affinity, if the lysine residue is methylated. In a peptide binding assay, the MBT domain of L3MBTL1–4 had a *K_D_* value between low and mid-micromolar range for histones, similar to other MBT-containing proteins, such as SCMH1, SFMBT1, and SMFBT2, that also have low affinity for histones (39). These results suggest that the poor affinity between the MBT domain and histones may be due to experimental difficulties, or histones are not the true substrate of these domains.

We show DNMT1K142me1 as a new binding partner for the MBT domain of PHF20L1, and one of the functions of this interaction is to protect the enzyme from degradation. The amino terminus of DNMT1 is susceptible to proteolytic degradation (40). Crystallography of DNMT1 suggests an unstructured amino terminus, suggesting poor packing of the protein, thus allowing proteases to bind and degrade this protein. Also, DNMT1 without the amino terminus is poor at targeting the replication foci because the PCNA binding site on DNMT1 lies between amino acids 162 and 174. Therefore, the presence of the amino terminus in DNMT1 is crucial for DNA methylation inheritance. It is plausible that PHF20L1a provides a dual function to DNMT1, with binding of PHF20L1a 1) masking the amino terminus region, thus preventing proteolysis, and 2) targeting DNMT1 to chromatin, using its PHD as a tether. Because the MBT domain selectively recognizes mono- and dimethyllysine and has been shown to function as a gene repressor, a functional cooperation with DNMT1 will contribute to the robust repressive state of the chromatin and facilitate DNA methylation. Colocalization of DNMT1 and PHF20L1a at the perinucleolar space in the presence of SET7, which catalyzes Lys-142 methylation, supports the above hypothesis. Mammalian perinucleolar space is the anchor to chromatin and represents inactive X allele and ribosomal RNA genes (41, 42). Ribosomal RNA genes are regulated by DNA methylation, and DNMT1 may be the predominant enforcer of gene regulation. Indeed, in DNMT1 null cells, rRNA gene methylation is abolished, resulting in aberrant expression (43). In a previous report, we have demonstrated that overexpression of SET7 depletes DNMT1, resulting in demethylation of rRNA genes (27).

Another intriguing observation concerns the presence of the other PHF20L1 isoforms, PHF20L1b and PHF20L1c, on chromatin, albeit at much lower levels when compared with PHF20L1a. This suggests that the MBT and the Tudor domain are essential for chromatin loading independent of the PHD. The Tudor domain has been described to bind strongly to di- or trimethylated histones, so it would be interesting to test the binding affinity of this domain toward histones or other non-histone proteins, such as p53.

Lysine residues on proteins can be methylated or acetylated. Methylation and acetylation of proteins may have different biological functions. In general, acetylated histones are predictors of transcriptionally active genes. Similarly, acetylation of p53 is responsible for transcriptional activation, and methylation enhances or suppresses p53 transcriptional activity, depending on the methylation site. Furthermore, in another study, the Tudor domain in PHF20 recognized p53 dimethylated at Lys-370 or Lys-382, and a homodimeric form of this Tudor domain could associate with the two dimethylated sites on p53 with enhanced affinity binding specificity, suggesting a multivalent interaction. This physical association between PHF20 and p53 promotes stabilization and activation of p53 by reducing Mdm2-mediated p53 ubiquitination and degradation (33). Indeed, a recent report has suggested that DNMT1 undergoes acetylation by Tip60, leading to its degradation (38). In the same report, the authors have suggested that acetylated DNMT1 is a substrate for the E3 ubiquitin ligase, UHRF1, a well known binding partner of DNMT1, leading to its proteasomal degradation. Indeed, DNMT1 can be stabilized by HDAC1 and destabilized by Tip60, suggesting that deacetylation and acetylation can be a major pathway for DNMT1 stability. HDAC1, which can deacetylate lysine residues, is a known binder of DNMT1, and this interaction could facilitate the methylation of DNMT1 by SET7. Methylated DNMT1 can then interact with PHF20L1a and remain stabilized during the cell cycle, and this may play an important role in epigenome silencing. Furthermore, PHF20L1 may play a vital role in epigenetic inheritance. Reduction of normal endogenous levels of PHF20L1 may influence aberrant DNA methylation and genomic instability. Therefore, the methyllysine reader PHF20L1 is an important reader of DNMTK142me1 that facilitates maintenance of this methyltransferase.

## Footnotes

3 The abbreviations and trivial names used are:

5mC: 5-methylcytosine
PCNA: proliferative cell nuclear antigen
DNMT1K142me1: DNMT1 lysine 142 monomethylated
5-aza-CdR: 5-aza-2′-deoxycytidine
MBT: malignant brain tumor
SET7: SET domain containing lysine methyltransferase 7
UNC1215: 2-phenylamino-1,4-bis(4-(pyrrolidinyl)piperidinyl)benzamide
MG132: *N*-(benzyloxycarbonyl)leucinylleucinylleucinal
CADOR: chromatin-associated domain array.

## References

[1] Kim J. K. Samaranayake M. Pradhan S. (2009) Epigenetic mechanisms in mammals. Cell Mol. Life Sci. 66, 596–612 18985277 10.1007/s00018-008-8432-4 PMC2780668

[2] Kinney S. R. Pradhan S. (2011) in Modifications of Nuclear DNA and Its Regulatory Proteins (Cheng X. Blumenthal R., eds) pp. 311–333, Elsevier Inc., London, UK

[3] Jones P. A. (2012) Functions of DNA methylation. Islands, start sites, gene bodies and beyond. Nat. Rev. Genet. 13, 484–492 22641018 10.1038/nrg3230

[4] Lister R. Pelizzola M. Dowen R. H. Hawkins R. D. Hon G. Tonti-Filippini J. Nery J. R. Lee L. Ye Z. Ngo Q. M. Edsall L. Antosiewicz-Bourget J. Stewart R. Ruotti V. Millar A. H. Thomson J. A. Ren B. Ecker J. R. (2009) Human DNA methylomes at base resolution show widespread epigenomic differences. Nature 462, 315–322 19829295 10.1038/nature08514 PMC2857523

[5] Chen T. Li E. (2006) Establishment and maintenance of DNA methylation patterns in mammals. in DNA Methylation: Basic Mechanisms (Doerfler W. Böhm P., eds) pp. 179–201, Springer, Berlin 10.1007/3-540-31390-7_6 16570848

[6] Ooi S. K. Qiu C. Bernstein E. Li K. Jia D. Yang Z. Erdjument-Bromage H. Tempst P. Lin S. P. Allis C. D. Cheng X. Bestor T. H. (2007) DNMT3L connects unmethylated lysine 4 of histone H3 to *de novo* methylation of DNA. Nature 448, 714–717 17687327 10.1038/nature05987 PMC2650820

[7] Suetake I. Shinozaki F. Miyagawa J. Takeshima H. Tajima S. (2004) DNMT3L stimulates the DNA methylation activity of Dnmt3a and Dnmt3b through a direct interaction. J. Biol. Chem. 279, 27816–27823 15105426 10.1074/jbc.M400181200

[8] Ehrlich M. (2006) Cancer-linked DNA hypomethylation and its relationship to hypermethylation. in DNA Methylation: Development, Genetic Disease and Cancer (Doerfler W. Böhm P., eds) pp. 251–274, Springer, Berlin 10.1007/3-540-31181-5_12 16909914

[9] Karpf A. R. Matsui S. (2005) Genetic disruption of cytosine DNA methyltransferase enzymes induces chromosomal instability in human cancer cells. Cancer Res. 65, 8635–8639 16204030 10.1158/0008-5472.CAN-05-1961

[10] Leonhardt H. Page A. W. Weier H. U. Bestor T. H. (1992) A targeting sequence directs DNA methyltransferase to sites of DNA replication in mammalian nuclei. Cell 71, 865–873 1423634 10.1016/0092-8674(92)90561-p

[11] Chuang L. S. Ian H. I. Koh T. W. Ng H. H. Xu G. Li B. F. (1997) Human DNA-(cytosine-5) methyltransferase-PCNA complex as a target for p21WAF1. Science 277, 1996–2000 9302295 10.1126/science.277.5334.1996

[12] Bostick M. Kim J. K. Estève P. O. Clark A. Pradhan S. Jacobsen S. E. (2007) UHRF1 plays a role in maintaining DNA methylation in mammalian cells. Science 317, 1760–1764 17673620 10.1126/science.1147939

[13] Guan D. Factor D. Liu Y. Wang Z. Kao H. Y. (2013) The epigenetic regulator UHRF1 promotes ubiquitination-mediated degradation of the tumor-suppressor protein promyelocytic leukemia protein. Oncogene 32, 3819–3828 22945642 10.1038/onc.2012.406 PMC3578017

[14] Citterio E. Papait R. Nicassio F. Vecchi M. Gomiero P. Mantovani R. Di Fiore P. P. Bonapace I. M. (2004) Np95 is a histone-binding protein endowed with ubiquitin ligase activity. Mol. Cell. Biol. 24, 2526–2535 14993289 10.1128/MCB.24.6.2526-2535.2004 PMC355858

[15] Li L. H. Olin E. J. Buskirk H. H. Reineke L. M. (1970) Cytotoxicity and mode of action of 5-azacytidine on L1210 leukemia. Cancer Res. 30, 2760–2769 5487063

[16] Stresemann C. Brueckner B. Musch T. Stopper H. Lyko F. (2006) Functional diversity of DNA methyltransferase inhibitors in human cancer cell lines. Cancer Res. 66, 2794–2800 16510601 10.1158/0008-5472.CAN-05-2821

[17] Santi D. V. Norment A. Garrett C. E. (1984) Covalent bond formation between a DNA-cytosine methyltransferase and DNA containing 5-azacytosine. Proc. Natl. Acad. Sci. U.S.A. 81, 6993–6997 6209710 10.1073/pnas.81.22.6993 PMC392062

[18] Jüttermann R. Li E. Jaenisch R. (1994) Toxicity of 5-aza-2′-deoxycytidine to mammalian cells is mediated primarily by covalent trapping of DNA methyltransferase rather than DNA demethylation. Proc. Natl. Acad. Sci. U.S.A. 91, 11797–11801 7527544 10.1073/pnas.91.25.11797 PMC45322

[19] Ghoshal K. Datta J. Majumder S. Bai S. Kutay H. Motiwala T. Jacob S. T. (2005) 5-Aza-deoxycytidine induces selective degradation of DNA methyltransferase 1 by a proteasomal pathway that requires the KEN box, bromo-adjacent homology domain, and nuclear localization signal. Mol. Cell. Biol. 25, 4727–4741 15899874 10.1128/MCB.25.11.4727-4741.2005 PMC1140649

[20] Yang X. D. Lamb A. Chen L. F. (2009) Methylation, a new epigenetic mark for protein stability. Epigenetics 4, 429–433 19829087 10.4161/epi.4.7.9787

[21] Black J. C. Van Rechem C. Whetstine J. R. (2012) Histone lysine methylation dynamics. Establishment, regulation, and biological impact. Mol. Cell 48, 491–507 23200123 10.1016/j.molcel.2012.11.006 PMC3861058

[22] Lachner M. O'Carroll D. Rea S. Mechtler K. Jenuwein T. (2001) Methylation of histone H3 lysine 9 creates a binding site for HP1 proteins. Nature 410, 116–120 11242053 10.1038/35065132

[23] Wysocka J. Swigut T. Milne T. A. Dou Y. Zhang X. Burlingame A. L. Roeder R. G. Brivanlou A. H. Allis C. D. (2005) WDR5 associates with histone H3 methylated at K4 and is essential for H3 K4 methylation and vertebrate development. Cell 121, 859–872 15960974 10.1016/j.cell.2005.03.036

[24] Kim J. Daniel J. Espejo A. Lake A. Krishna M. Xia L. Zhang Y. Bedford M. T. (2006) Tudor, MBT and chromo domains gauge the degree of lysine methylation. EMBO Rep. 7, 397–403 16415788 10.1038/sj.embor.7400625 PMC1456902

[25] Vermeulen M. Eberl H. C. Matarese F. Marks H. Denissov S. Butter F. Lee K. K. Olsen J. V. Hyman A. A. Stunnenberg H. G. Mann M. (2010) Quantitative interaction proteomics and genome-wide profiling of epigenetic histone marks and their readers. Cell 142, 967–980 20850016 10.1016/j.cell.2010.08.020

[26] Bonasio R. Lecona E. Reinberg D. (2010) MBT domain proteins in development and disease. Semin. Cell Dev. Biol. 21, 221–230 19778625 10.1016/j.semcdb.2009.09.010 PMC3772645

[27] Estève P. O. Chin H. G. Benner J. Feehery G. R. Samaranayake M. Horwitz G. A. Jacobsen S. E. Pradhan S. (2009) Regulation of DNMT1 stability through SET7-mediated lysine methylation in mammalian cells. Proc. Natl. Acad. Sci. U.S.A. 106, 5076–5081 19282482 10.1073/pnas.0810362106 PMC2654809

[28] Estève P. O. Chang Y. Samaranayake M. Upadhyay A. K. Horton J. R. Feehery G. R. Cheng X. Pradhan S. (2011) A methylation and phosphorylation switch between an adjacent lysine and serine determines human DNMT1 stability. Nat. Struct. Mol. Biol. 18, 42–48 21151116 10.1038/nsmb.1939 PMC3048033

[29] Andrews N. C. Faller D. V. (1991) A rapid micropreparation technique for extraction of DNA-binding proteins from limiting numbers of mammalian cells. Nucleic Acids Res. 19, 2499 2041787 10.1093/nar/19.9.2499 PMC329467

[30] Estève P. O. Chin H. G. Smallwood A. Feehery G. R. Gangisetty O. Karpf A. R. Carey M. F. Pradhan S. (2006) Direct interaction between DNMT1 and G9a coordinates DNA and histone methylation during replication. Genes Dev. 20, 3089–3103 17085482 10.1101/gad.1463706 PMC1635145

[31] Pradhan S. Kim G. D. (2002) The retinoblastoma gene product interacts with maintenance human DNA (cytosine-5) methyltransferase and modulates its activity. EMBO J. 21, 779–788 11847125 10.1093/emboj/21.4.779 PMC125847

[32] Pradhan S. Bacolla A. Wells R. D. Roberts R. J. (1999) Recombinant human DNA (cytosine-5) methyltransferase. I. Expression, purification, and comparison of *de novo* and maintenance methylation. J. Biol. Chem. 274, 33002–33010 10551868 10.1074/jbc.274.46.33002

[33] Cui G. Park S. Badeaux A. I. Kim D. Lee J. Thompson J. R. Yan F. Kaneko S. Yuan Z. Botuyan M. V. Bedford M. T. Cheng J. Q. Mer G. (2012) PHF20 is an effector protein of p53 double lysine methylation that stabilizes and activates p53. Nat. Struct. Mol. Biol. 19, 916–924 22864287 10.1038/nsmb.2353 PMC3454513

[34] Espejo A. Côté J. Bednarek A. Richard S. Bedford M. T. (2002) A protein-domain microarray identifies novel protein-protein interactions. Biochem. J. 367, 697–702 12137563 10.1042/BJ20020860 PMC1222921

[35] Fortschegger K. Shiekhattar R. (2011) Plant homeodomain fingers form a helping hand for transcription. Epigenetics 6, 4–8 20818169 10.4161/epi.6.1.13297 PMC3044459

[36] Rock K. L. Gramm C. Rothstein L. Clark K. Stein R. Dick L. Hwang D. Goldberg A. L. (1994) Inhibitors of the proteasome block the degradation of most cell proteins and the generation of peptides presented on MHC class I molecules. Cell 78, 761–771 8087844 10.1016/s0092-8674(94)90462-6

[37] James L. I. Barsyte-Lovejoy D. Zhong N. Krichevsky L. Korboukh V. K. Herold J. M. MacNevin C. J. Norris J. L. Sagum C. A. Tempel W. Marcon E. Guo H. Gao C. Huang X. P. Duan S. Emili A. Greenblatt J. F. Kireev D. B. Jin J. Janzen W. P. Brown P. J. Bedford M. T. Arrowsmith C. H. Frye S. V. (2013) Discovery of a chemical probe for the L3MBTL3 methyllysine reader domain. Nat. Chem. Biol. 9, 184–191 23292653 10.1038/nchembio.1157 PMC3577944

[38] Du Z. Song J. Wang Y. Zhao Y. Guda K. Yang S. Kao H. Y. Xu Y. Willis J. Markowitz S. D. Sedwick D. Ewing R. M. Wang Z. (2010) DNMT1 stability is regulated by proteins coordinating deubiquitination and acetylation-driven ubiquitination. Sci. Signal. 3, ra80 21045206 10.1126/scisignal.2001462 PMC3116231

[39] Nady N. Krichevsky L. Zhong N. Duan S. Tempel W. Amaya M. F. Ravichandran M. Arrowsmith C. H. (2012) Histone recognition by human malignant brain tumor domains. J. Mol. Biol. 423, 702–718 22954662 10.1016/j.jmb.2012.08.022

[40] Bestor T. H. Ingram V. M. (1985) Growth-dependent expression of multiple species of DNA methyltransferase in murine erythroleukemia cells. Proc. Natl. Acad. Sci. U.S.A. 82, 2674–2678 3857609 10.1073/pnas.82.9.2674 PMC397627

[41] McStay B. Grummt I. (2008) The epigenetics of rRNA genes. From molecular to chromosome biology. Annu. Rev. Cell Dev. Biol. 24, 131–157 18616426 10.1146/annurev.cellbio.24.110707.175259

[42] Zhang L. F. Huynh K. D. Lee J. T. (2007) Perinucleolar targeting of the inactive X during S phase. Evidence for a role in the maintenance of silencing. Cell 129, 693–706 17512404 10.1016/j.cell.2007.03.036

[43] Espada J. Ballestar E. Santoro R. Fraga M. F. Villar-Garea A. Németh A. Lopez-Serra L. Ropero S. Aranda A. Orozco H. Moreno V. Juarranz A. Stockert J. C. Längst G. Grummt I. Bickmore W. Esteller M. (2007) Epigenetic disruption of ribosomal RNA genes and nucleolar architecture in DNA methyltransferase 1 (Dnmt1) deficient cells. Nucleic Acids Res. 35, 2191–2198 17355984 10.1093/nar/gkm118 PMC1874631

